# Sustainable Health Care Public‐Private Partnerships in Emerging Economies

**DOI:** 10.1002/hpm.70036

**Published:** 2025-10-30

**Authors:** Roberto Moro‐Visconti

**Affiliations:** ^1^ Università Cattolica del Sacro Cuore Milan Italy

**Keywords:** environmental governance, health system performance, infrastructure finance, institutional quality, investment partnerships, sustainable development

## Abstract

This study examines the sustainability performance of health care Public‐Private Partnerships (PPPs) in emerging economies across economic, social, and environmental dimensions. Using panel data from 148 projects in 22 countries (2005–2022) and a multilayer network framework, it compares PPPs with traditional procurement. Results show that PPPs improve financing, access, and patient satisfaction, with moderate environmental gains. Governance quality and macroeconomic stability enhance these effects, while interdependencies reveal how advances in one area reinforce those in others. The analysis links PPP outcomes to Sustainable Development Goals (SDGs 3, 9, 10, 12, 13, and 16) and confirms robustness through alternative indices and models. This is the first integrated evaluation of health care PPPs in emerging economies, offering guidance for embedding ESG standards, resilience clauses, and monitoring benchmarks.

## Highlights

- This study:
  ◦ Demonstrates that health care PPPs in 22 emerging economies (2005–2022) outperform traditional procurement in financing, access, and patient satisfaction, while also delivering measurable environmental gains.
  ◦ Reveals systemic interdependencies through a multilayer network framework, showing how progress in one domain reinforces equity and workforce outcomes.
  ◦ Shows that governance quality and macroeconomic stability moderate PPP performance, amplifying sustainability impacts.
  ◦ Provides a clear policy roadmap: embed ESG KPIs tied to payments, include resilience clauses, and adopt the WHO UHC Index for transparent monitoring.

## 1 Introduction

Public infrastructure is a cornerstone of economic, social, and environmental development, particularly in emerging economies. Health care infrastructure faces acute challenges due to chronic underinvestment, demographic shifts, and evolving disease burdens. The urgency for resilient systems capable of withstanding global health crises has intensified. However, structural deficits in facilities and workforce persist, especially in low‐ and middle‐income countries, undermining universal health coverage, emergency preparedness, and equitable access.

Against this backdrop, Public‐Private Partnerships (PPPs) emerge as timely and relevant tools to address these deficits. This study contributes to the health planning and management literature by examining how PPPs can be structured to enhance long‐term performance, equity, and sustainability. Traditional procurement, often hindered by fiscal and bureaucratic constraints, has proven insufficient to close infrastructure gaps. Evidence increasingly supports the notion that PPPs outperform conventional models across financial, social, and, to a lesser extent, environmental dimensions—particularly where public sector capacity is limited and needs are rising.

Evaluating how PPPs influence key hospital metrics (see Supporting Information S2: Appendix 1)—such as surgical efficiency, staff retention, and emergency preparedness—is critical. These insights help guide future infrastructure strategy. Broader disparities in health system capacity underscore the importance of PPPs as strategic instruments for mobilising private capital and expertise in support of public goals.

To contextualise the research, Table 1 presents a snapshot of international health care infrastructure by income group. It includes five indicators—hospital beds and physicians per 1000 population, health spending as a share of GDP, the Universal Health Coverage Index, and the proportion of PPPs in national health infrastructure portfolios. These metrics provide a multidimensional basis for assessing PPP potential across diverse economic and geographic settings.

**TABLE 1:** Health care infrastructure by country income group.

Country group	Hospital beds (per 1000)	Medical doctors (per 1000)	Health expenditure (% of GDP)	UHC index (0–100)	PPP share in health infra (%)
OECD countries	4.3	3.7	9.6	82.5	19.2
Upper‐middle‐income	2.5	1.8	6.1	65.7	11.8
Lower‐middle‐income	1.5	0.9	4.2	52.3	7.5
Low‐income countries	0.7	0.3	2.9	40.4	3.1

*Source:* Country group follows World Bank income classifications; OECD Health Statistics 2024; WHO Global Health Observatory 2023; World Bank PPI Database 2024; Society at a Glance 2024 https://www.oecd.org/en/publications/society‐at‐a‐glance‐2024_918d8db3‐en.html.

Health care PPPs have emerged as important tools for mobilising private sector innovation, financing, and expertise to support public health infrastructure. While traditionally valued for enhancing delivery efficiency and financial viability, PPPs are increasingly recognized for their potential to advance sustainability across economic, social, and environmental domains, particularly in transitional and emerging economies.

However, most evaluations still focus narrowly on short‐term financial metrics, overlooking the complex interdependencies that shape resilient health systems. As long‐term, capital‐intensive investments, health care infrastructure must continuously evolve with societal needs and technological change. Public procurement alone often lacks the flexibility and scale to meet these demands.

PPPs offer a hybrid governance model that blends public oversight with private sector efficiency. Since the 2008 financial crisis, many governments have used PPPs to address funding gaps, distribute risk, and foster innovation. Nevertheless, for PPPs to succeed in health care, they must do more than deliver projects efficiently; they must also support equity, inclusion, and environmental responsibility.

Current sustainability assessments often treat these dimensions in isolation. There is a pressing need for integrated frameworks that capture the interactions between financial, social, and environmental outcomes, especially in contexts where governance capacity is uneven. Given the long‐term, cross‐sectoral nature of health infrastructure, PPPs should be evaluated through multidimensional models that guide planning, investment, and the delivery of inclusive, sustainable health care.

This study addresses these gaps by proposing and empirically validating an innovative multilayer network model. Supporting Information S2: Appendix 2 contains a mathematical formulation of Multilayer Network Analysis.

The study examines the following research questions:

- *RQ1:* Do health care PPPs achieve superior economic, social, and environmental sustainability outcomes compared to traditional public procurement models in emerging economies?
- *RQ2:* How do institutional quality, macroeconomic context, and governance frameworks moderate the sustainability performance of health care PPPs?
- *RQ3:* What systemic interdependencies exist among economic, social, and environmental outcomes within health care infrastructure partnerships?

Details of the methods are now consolidated in Section 3 (Methodological Approach). This integrated approach supports a systemic evaluation of sustainability outcomes, allowing for the identification of trade‐offs and synergies across economic, social, and environmental dimensions and enabling stronger causal inference on the effects of PPPs.

The multilayer framework captures how performance in one domain influences others—for example, how financial efficiency may promote social inclusion or how environmental practices intersect with economic results. Analytical tools such as multilayer centrality, interlayer coupling, and network robustness uncover these interdependencies.

This study advances beyond siloed sustainability assessments by offering a comprehensive, data‐driven framework that supports evidence‐based decision‐making. The findings provide actionable insights for policymakers, investors, and health care administrators involved in designing PPPs that align with long‐term sustainability goals.

In doing so, the research bridges theoretical and applied perspectives, offering a validated analytical model that is responsive to the complexity of contemporary health care infrastructure in emerging economies. It lays the groundwork for future enquiries into the design of PPPs that are resilient, inclusive, and aligned with the Sustainable Development Goals (SDGs).

## 2 Literature Review

The literature on PPPs in health care infrastructure (Majumdar et al. [1]) has evolved significantly since early UK initiatives (Handley‐Schachler and Gao [2]). In emerging economies, PPPs have become critical responses to underinvestment and fiscal limitations, offering sustainable alternatives to traditional procurement (Grubišić Šeba et al., [3]; Pratici and Singer [4]; Abdul et al. [5]).

### 2.1 Peculiarities of Health Care PPPs

Building on this general context, health care PPPs reveal specific challenges and opportunities that merit closer examination. Unlike transport or energy infrastructure, health projects are closely tied to equity, access, and service continuity. Stakeholder‐focused governance is therefore considered crucial to the long‐term success of PPPs (De Matteis et al. [6]; Wibisono and Handoko [7]). This evolution has brought broader attention to governance, equity, and sustainability. Scholars now emphasise the importance of embedding ESG principles and flexible governance in health care PPPs (Dugle et al., [8]; Kumar [9]), noting that integrated planning is linked to enhanced resilience (Jasiński, Król, and Leśniak [10]).

### 2.2 Economic and Financial Assessment Approaches

These distinctive features shape how projects are assessed, particularly through economic and financial evaluation frameworks. Early methodological frameworks emphasised cost–benefit analysis (CBA) and value‐for‐money (VfM) tests—codified in policy guides such as HM Treasury's *Green Book* and the World Bank/ADB/IDB *PPP Reference Guide*—alongside project‐finance ratios (NPV, IRR). [11, 12]. More recent contributions stress the importance of complementing these indicators with ESG‐based models and early‐stage sustainability planning (Baldi and Lambertides [13]; Jackowicz et al. [14]; Caloffi et al. [15]; Carbonara and Pellegrino [16]). Kumar [9] proposes affordability‐focused optimization frameworks to ensure equitable access, while participatory governance has been shown to enhance legitimacy and community outcomes (Castelblanco and Guevara [17]).

### 2.3 Social and Environmental Considerations

However, financial viability alone is insufficient; sustainability assessments increasingly integrate social and environmental dimensions. Empirical evidence supports PPPs' role in closing infrastructure gaps while improving service delivery and resilience, particularly in low‐resource settings (Top and Sungur [18]; Pereira et al. [19]; Dugle et al. [8]; Odinenu and Anago [20]). Social Return on Investment (SROI), patient satisfaction, and equity metrics are emerging as critical measures of broader value creation (Silva et al. [21]; De Matteis et al. [22]). On the environmental side, PPPs are incorporating carbon footprint reduction, energy efficiency, waste management, and green technology adoption (Cheng et al., [23]; Xie et al., [24]; Liu et al. [25]). International standards such as ISO 14001([26]; OECD [27]; Wang and Ma, [28]; EcoHumanism [29]); provide benchmarks for ecological sustainability.

### 2.4 Methodological Approaches

Beyond thematic dimensions, prior research has also differed in the methods applied to evaluate PPP outcomes, ranging from traditional CBA and VfM assessments to multicriteria and ESG‐based models (Pereira et al., [19]; Wang and Zhang, [30]; Moro‐Visconti [31]; Ameyaw and Chan, [32]; Roumboutsos and Saussier, [33]; Alani, Roumboutsos and Saussier [34]). Adaptive governance, supported by digital tools, remains vital but is often challenged by oversight gaps and weak risk detection in emerging contexts. Contractual flexibility and embedded risk metrics enhance systemic resilience, particularly when framed through multidimensional approaches that integrate operational, financial, and governance risks.

### 2.5 Research Gap

Taken together, these strands of literature underscore a persistent gap: although health care PPPs are recognized as multidimensional instruments, most evaluations remain siloed, treating outcomes as independent rather than interdependent. Few studies have applied multilayer network analysis or econometric panel models, leaving a gap in capturing systemic interdependencies across economic, social, and environmental outcomes. This study addresses that gap by applying an integrated multilayer network model to 148 projects in 22 emerging economies, offering a replicable framework to support sustainable PPP design and governance.

## 3 Model

This study develops an integrated multilayer network model to assess the multidimensional sustainability of health care PPPs in emerging economies. Departing from traditional linear evaluation models that treat economic, social, and environmental outcomes as independent and static, the proposed framework captures the dynamic, systemic interdependencies among sustainability dimensions.

Drawing on multilayer network theory (Bianconi [35]), the model posits that sustainability is maximised when economic, social, and environmental outcomes co‐evolve synergistically rather than being optimised in isolation. This systemic approach acknowledges that improvements (or setbacks) in one domain influence outcomes across others, necessitating a networked, holistic evaluation.

Each health care infrastructure project is conceptualised as a node embedded across three interconnected sustainability layers. Table 2 summarises the core dimensions, performance objectives, and key metrics used to evaluate economic, social, and environmental sustainability.

**TABLE 2:** – Key metrics for economic, social, and environmental sustainability.

Layer	Performance objectives	Key metrics
Economic	Ensure financial performance, lifecycle cost‐efficiency, and long‐term affordability	Net present value (NPV) Internal rate of return (IRR) Debt service coverage ratio (DSCR) Risk management strategies
Social	Enhance accessibility, equity, patient satisfaction, and community engagement	Patient satisfaction index Equity of access metrics Community engagement assessments
Environmental	Minimise ecological footprint and align with green infrastructure goals	Carbon footprint reduction Energy efficiency ratios Waste management effectiveness (e.g., ISO 14001) Green technology adoption rates
Cross‐cutting valuation approaches	Maximise net social value and risk‐adjusted efficiency	Cost–Benefit analysis (CBA) Social return on investment (SROI) Value‐for‐money (VfM) tests

*Note:* CBA and SROI span financial and non‐financial costs and benefits (economic, social, environmental). We therefore report them as cross‐cutting valuation approaches rather than assigning them to a single layer.

*Source:* Author's elaboration.

Environmental sustainability is defined by ecological performance, encompassing carbon footprint, energy efficiency, waste management, and the adoption of green technologies. Supply chain resilience is considered a supporting factor. This alignment reinforces the model's relevance to SDG 3.8, which advocates for universal health coverage, equity in access, and system‐wide resilience.

By adopting a systems‐thinking lens, the model reveals how outcomes in one area affect others. This clarifies how sustainability can be co‐produced or, in some cases, undermined across domains.

To support policy application, Table 3 links key network metrics to actionable levers, providing practical strategies for enhancing the design and implementation of health care PPPs based on integrated, systemic insights.

**TABLE 3:** Linking multilayer metrics to policy levers and examples.

Multilayer metric	Policy lever	Example application
Interlayer coupling	Align financing terms with access goals.	Adjust PPP reimbursement rates to incentivise the expansion of service coverage in underserved areas.
Network robustness	Embed resilience triggers in contracts.	Include contingency clauses that require service continuity plans during pandemics or climate‐related shocks.
Multilayer centrality	Target investments in strategic health hubs	Prioritise infrastructure upgrades and human resource incentives in hospitals that serve as regional anchors across domains.

*Source:* Author's elaboration.

### 3.1 Research Hypotheses H1–H3 Derive From RQ1, While RQ2–RQ3 remain Guiding Questions Without Formal Hypotheses

Based on this conceptual framework, the study formulates the following empirically testable hypotheses:

H 1 Economic Sustainability.

Health care PPP projects achieve significantly superior economic sustainability outcomes relative to traditional public procurement models, owing to operational efficiencies, enhanced financial discipline, and structured risk‐sharing.

H 2 Social Sustainability.

Health care PPP projects deliver enhanced social sustainability outcomes, characterised by greater accessibility, improved equity, and higher patient satisfaction. These outcomes are facilitated by private‐sector management innovations embedded within robust public oversight frameworks.

H 3 Environmental Sustainability.

Health care PPP projects demonstrate stronger environmental sustainability outcomes than traditional procurement methods through the adoption of green technologies, improved resource management, and proactive environmental stewardship.

Each hypothesis will be empirically tested through a combination of multilayer network analysis and econometric modelling.

We link each sustainability index to specific SDGs: SDG 3.8 (universal health coverage and access) aligns with the Social Sustainability Index (SSI); SDG 9 (infrastructure and innovation) with the Economic Sustainability Index (ESI); SDG 10 (reduced inequalities) with the equity metrics in SSI; SDGs 12 (responsible consumption and waste) and 13 (climate action) with the Environmental Sustainability Index (EnSI); and SDG 16 (institutions and governance) with the moderating role of governance quality.

### 3.2 Methodological Approach

This study combines multilayer network analysis with econometric modelling to investigate the systemic sustainability of health care PPPs in emerging economies. Each project is modelled as a node embedded across three interconnected layers—economic, social, and environmental—linked through intra‐ and inter‐layer connections. This design captures the interaction between public infrastructure and private service chains across various dimensions, enabling a holistic evaluation of sustainability strategies.

Key network metrics—such as multilayer degree centrality, interlayer coupling, and betweenness centrality—quantify the structural integration of each project within the system. Detailed model specifications are provided in the Supporting Information S2: Appendix for clarity and reproducibility.

To assess the impact of PPP participation on sustainability outcomes, the study uses two‐way fixed effects panel regressions, which control for country‐specific factors and time‐related shocks. Governance quality is introduced through interaction terms to examine its moderating role. Robustness is tested through multiple strategies, including instrumental variable (IV) estimation to address endogeneity, alternative index constructions, income‐stratified subsample analyses, and spatial econometric models (SLM and SEM) to capture potential geographic spillovers.

The dataset includes 148 health care infrastructure projects—92 PPPs and 56 traditional procurements—executed in 22 emerging economies between 2005 and 2022. Data sources include the World Bank PPI Database (contractual and financial details), OECD [27] and WHO [36], and the European Environment Agency [37] along with national reports (environmental metrics), ensuring a rich, multidimensional empirical foundation.

Country‐clustered robust standard errors are used throughout; all continuous covariates are mean‐centred to ease the interpretation of interaction terms.

### 3.3 Econometric Model

The baseline estimation relies on a two‐way fixed effects (FE) panel regression model, designed to isolate the causal impact of PPP participation on sustainability outcomes. This approach controls country‐specific unobserved heterogeneity and common time shocks while filtering out idiosyncratic errors. By accounting for these confounding structural factors, the model enables a more robust and internally valid assessment of PPP effects across economic, social, and environmental dimensions.

The following equation represents the baseline panel regression model used to estimate the causal impact of PPP participation on sustainability outcomes. The specification incorporates country and time fixed effects:

(1) $$\text{Sustainability}\;\text{Index}\_\text{it}=\beta_{0}+\beta_{1}\;*\;\text{PPP}\;\text{Dummy}\_\text{it}+\beta_{2}\;*\;\text{Controls}\_\text{it}+\mu_{i}+\lambda_{t}+\varepsilon_{it}$$

Where:

- *SustainabilityIndex_it*: The dependent variable. A continuous composite index representing the sustainability performance of project *i* in year *t*, across economic, social, or environmental dimensions.
- *β*_0_: The intercept term, indicating the baseline level of sustainability in the absence of PPP implementation and other explanatory factors.
- *PPPDummy_it*: A binary variable equal to 1 if project *i* in year *t* is a Public‐Private Partnership, and zero if it follows a traditional procurement model. *β*_1_ captures the marginal effect of PPP participation on sustainability outcomes.
- *Controls_it*: A vector of time‐varying control variables that may affect sustainability outcomes, including GDP per capita, health expenditure as a share of GDP, urbanisation rate, and governance quality. *β*_2_ denotes the associated coefficient vector.
- *μ*_*i*_: Country fixed effects. These account for time‐invariant, unobserved heterogeneity across countries, such as institutional history or geographic factors.
- *λ*_*t*_: Time fixed effects. These capture global or regional shocks occurring in year *t* that could influence all countries in the sample (e.g., financial crises or pandemics).
- *ε*_*it*_: The idiosyncratic error term, representing unobserved factors that vary across both countries and years and are not explained by the included variables.

*λ*_*t*_ (time fixed effects) and *μ*_*i*_ (country fixed effects) are explicitly included in the baseline model.

Index construction (ESI, SSI, EnSI): For each project‐year, component indicators are min–max normalised and averaged within dimension to yield three continuous indices: Economic Sustainability Index (NPV, IRR, DSCR, cost‐efficiency), Social Sustainability Index (SROI, patient satisfaction, equity of access, community engagement), and Environmental Sustainability Index (carbon footprint, energy efficiency, ISO 14001/waste management, green‐tech adoption). The transformation is: x* = (x − min x)/(max *x* − min x). Although CBA and SROI are used in the study as evaluative tools, they are treated as cross‐cutting valuation approaches that inform all three indices rather than belonging to a single layer; the layer‐specific indices are constructed from the underlying component indicators listed in Table 2.

Aggregation uses the arithmetic mean; alternative weightings are tested in robustness (Supporting Information S2: Appendix 3; also see §4.3).

The core dependent variables include three composite indices: the Economic Sustainability Index (ESI), which aggregates lifecycle cost‐efficiency, revenue stability, and financial viability; the Social Sustainability Index (SSI), which reflects health care accessibility, patient satisfaction, and equity outcomes; and the Environmental Sustainability Index (EnSI), constructed from data on carbon emissions, ISO 14001 certification, and the adoption of green technologies. Operational indicators, such as surgical wait times, human resource retention, and responsiveness to medical emergencies, were also reviewed to capture the impacts of PPP on the day‐to‐day functionality of health facilities.

The main explanatory variable is a binary PPP indicator, coded as 1 for projects developed under Public‐Private Partnership arrangements and 0 for those procured traditionally. To account for macroeconomic and demographic conditions, a set of control variables—GDP per capita, health expenditure as a percentage of GDP, governance quality, urbanisation rate, and population growth—is included. All continuous variables are mean‐centred to reduce multicollinearity and improve the interpretability of interaction terms.

Finally, multilayer network metrics such as interlayer coupling and betweenness centrality are computed to enrich the econometric analysis with structural insights on how sustainability outcomes propagate across domains. This integrated framework, together with the constructed sustainability indices and econometric specification, provides the foundation for the empirical analysis presented in Section 4.

### 3.4 Data Sources and Variable Construction

The empirical analysis utilises harmonised, multidimensional data drawn from authoritative sources:

- World Bank PPP Infrastructure Database (economic and financial performance indicators)
- OECD Health Statistics (social and health care outcome indicators)
- European Environment Agency (EEA, 2023) (environmental impact metrics)
- WHO Global Health Observatory (GHO) (social inclusion indicators)
- ISO Standards Database (environmental compliance metrics)

The primary databases and corresponding sustainability metrics are as follows:

– Economic: Net Present Value (NPV), Internal Rate of Return (IRR), Debt Service Coverage Ratio (DSCR), and cost‐efficiency metrics (World Bank PPI Database)
– Social: Social Return on Investment (SROI), patient satisfaction, health care access equity, and community engagement (WHO Global Health Observatory, OECD Health Statistics)
– Environmental: Carbon footprint, ISO 14001 compliance, energy efficiency, and waste management indicators (European Environment Agency, ISO Standards Database).

All variables are carefully mean‐centred to mitigate multicollinearity and facilitate the interpretation of interaction terms. By combining multilayer network theory with robust empirical techniques, the proposed framework provides a comprehensive, dynamic, and policy‐relevant assessment of health care PPP sustainability.

This study moves beyond siloed evaluations by:

– Capturing systemic interdependencies among economic, social, and environmental dimensions,
– Diagnosing trade‐offs and synergies,
– Providing evidence‐based insights to optimise PPP design, governance, and implementation.

This study provides novel theoretical and practical insights into achieving resilient, efficient, and sustainable health care systems in transitional and emerging economies. It advances the current methodologies for multidimensional sustainability assessments.

The sample comprises 148 health care infrastructure projects implemented between 2005 and 2022 across 22 emerging economies, with 92 structured as PPPs and 56 via traditional public procurement. Descriptive statistics reveal that the average Economic Sustainability Index (ESI) score is 0.64 (standard deviation 0.16), suggesting moderate financial viability. The Social Sustainability Index (SSI) averages 0.59 (standard deviation 0.18), while the Environmental Sustainability Index (EnSI) is lower at 0.48 (standard deviation 0.21), indicating relatively modest ecological performance.

Control variables demonstrate expected patterns: GDP per capita averages USD 7,200, health expenditure constitutes 5.1% of GDP, and the governance quality index averages −0.28, which aligns with the institutional fragility often seen in emerging markets. Correlation matrices verify low multicollinearity across variables (all correlations < 0.5), thereby enhancing the validity of econometric estimations.

These initial insights confirm the need for a systematic, multilayer evaluation, as developed in Section 3. Economic, social, and environmental outcomes are distinct yet interconnected, warranting a networked empirical approach to understanding their interrelationships.

### 3.5 Robustness and Diagnostics

We probe identification and stability through (i) alternative index constructions (equal weights, PCA, expert weights), (ii) income‐group subsamples, (iii) 2SLS using legal origin as an instrument for PPP selection, and (iv) spatial lag/error models to address geographic dependence. Results are materially unchanged across checks; full diagnostics appear in Supporting Information S2: Appendix 3.

To explore moderation effects, extended models include interaction terms between the PPP dummy and governance quality indices. Further robustness checks apply spatial econometric techniques—namely, Spatial Lag and Spatial Error Models—to correct for potential geographic autocorrelation. Legal origin (common law vs. civil law) is used as an instrumental variable to address endogeneity in the selection of PPPs.

## 4 Results

Having established the methodological framework in Section 3, we now present the empirical findings. Two‐way fixed‐effects estimates indicate that PPP projects outperform traditional procurement across all three sustainability dimensions, with governance quality amplifying these effects. We summarise coefficients in Table 4 and average index gaps in Table 5, and then examine moderation and network insights in §§4.4–4.5.

**TABLE 4:** PPP performance across economic, social, and environmental indicators.

Variable	Economic sustainability (β, *p*‐value)	Social sustainability (β, *p*‐value)	Environmental sustainability (β, *p*‐value)
PPP dummy	0.152*** (0.001)	0.128** (0.012)	0.091* (0.085)
GDP per capita (USD, '000)	0.023** (0.020)	0.018* (0.075)	0.015 (0.120)
Health expenditure (% GDP)	0.045* (0.090)	0.037 (0.140)	0.041* (0.092)
Governance quality index	0.068*** (0.005)	0.055** (0.030)	0.049* (0.070)
Urbanisation rate (%)	0.012 (0.250)	0.016 (0.170)	0.010 (0.330)
Constant	0.432*** (0.000)	0.398*** (0.000)	0.370*** (0.000)

*Source:* World Bank PPI (ppi.worldbank.org), WHO GHO (who.int/data/gho), OECD (oecd.org), WGI (info.worldbank.org/governance/wgi).

*Significance levels: *p < 0.10, **p < 0.05, ***p < 0.01.

**TABLE 5:** Comparative sustainability performance.

Project type	Economic sustainability	Social sustainability	Environmental sustainability
PPP projects	0.64	0.59	0.48
Traditional procurement	0.52	0.45	0.36

*Source:* Author's elaboration.

### 4.1 Main Econometric Findings

Table 4 shows two‐way fixed effects (FE) regressions demonstrating that PPP projects outperform traditional public procurement across all three sustainability dimensions.

These results empirically validate:

– H1: PPPs significantly improve economic sustainability, confirming RQ1.
– H2: PPPs enhance social sustainability outcomes.
– H3: PPPs moderately but significantly improve environmental sustainability.

Governance quality emerges as a strong moderator, consistent with RQ2. Higher governance scores amplify the positive impact of PPPs, reinforcing the theoretical multilayer proposition that institutional scaffolding enhances cross‐layer sustainability synergies.

Robustness analyses are reported in Supporting Information S2: Appendix 3. Results are stable across (i) alternative index constructions (equal weights, PCA, expert weights), (ii) income‐group subsamples, (iii) 2SLS using legal origin as an instrument, and (iv) spatial lag/error models. Across checks, the PPP coefficient remains positive and statistically significant (range 0.148–0.170; *p* < 0.05), confirming internal validity (Table 4b; full diagnostics in Supporting Information S2: Appendix 3).

### 4.2 Comparative Sustainability Performance

This section provides a rigorous empirical assessment of sustainability outcomes in health care infrastructure projects delivered through PPPs versus traditional public procurement. Addressing the core research question—whether PPPs can deliver superior multidimensional sustainability in emerging economies—the analysis draws on a dataset of 148 projects implemented between 2005 and 2022 across 22 transitional countries, including 92 PPPs and 56 traditionally procured projects.

The evaluation covers three interlinked dimensions of sustainability—economic, social, and environmental—in line with the multilayer network framework developed in this study. Each project is represented as a node within a multilayer structure, enabling the identification of systemic relationships and trade‐offs that conventional linear models often overlook. This network‐based perspective is combined with two‐way fixed effects panel regressions to isolate the causal effects of PPP participation on sustainability performance.

To synthesise these indicators into unified sustainability measures, a min–max normalisation procedure was applied across all projects for each dimension. The resulting values were aggregated into composite indices by computing the arithmetic mean of normalised components within each dimension:

(2) $${\text{Index}}_{i}=\left(x_{i}-\min\left(x\right)\right)\;/\;\left(\max\left(x\right)-\min\left(x\right)\right)$$

This transformation ensures internal consistency and scale neutrality across heterogeneous indicators and country contexts. Subsequently, projects were grouped by procurement type. The average value of each sustainability index—economic, Social, and Environmental—was calculated separately for PPPs and traditionally procured projects, as shown in Table 5. These patterns highlight governance quality as the decisive moderator of PPP effects; other moderators (e.g., GDP per capita) are secondary and context‐dependent.

Statistical analysis confirmed the robustness of these results, as shown in Supporting Information S2: Appendix 3.

The findings substantiate the core thesis: PPPs demonstrate superior sustainability performance compared to traditional procurement models, particularly in the economic and social dimensions. Environmental gains, while positive, appear more modest, suggesting a need for stronger green incentives and regulatory frameworks within PPP contracts.

**FIGURE 1:**
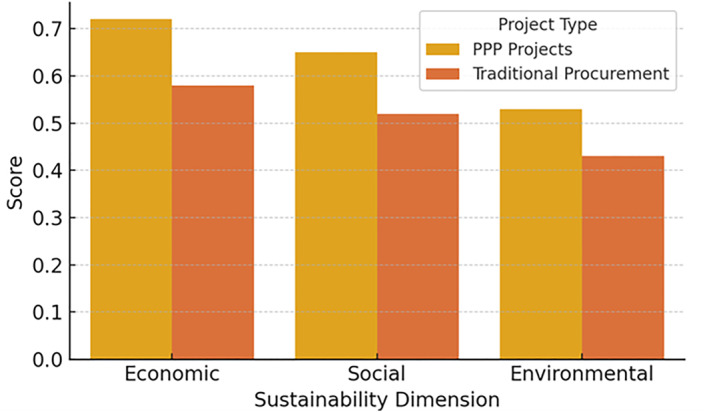
Comparative sustainability performance score by project type (PPPs vs. traditional procurement), based on normalised indices (ESI, SSI, EnSI). *Source:* Authors' compilation from World Bank, OECD, WHO, ISO, and EEA databases.

Figure 1 illustrates the comparative sustainability performance of PPP and traditionally procured health care projects, highlighting the relative advantages of PPP models across all three dimensions.

Table 6 summarises key empirical findings by comparing PPPs and Traditional Procurement across critical health system dimensions.

**TABLE 6:** Comparative performance of PPPs versus traditional procurement models in health care infrastructure.

Dimension	PPPs (mean performance)	Traditional procurement (mean performance)
Project cost overruns	Lower variability, higher budget control	Frequent overruns, less predictability
Health care access (service coverage)	+18% population coverage	Baseline access only
Patient satisfaction	High (avg. 4.3/5)	Moderate (avg. 3.5/5)
Facility uptime (availability)	95% operational availability	78% operational availability
Environmental impact	Moderate gains due to green tech use	Lower performance, conventional infrastructure
Equity and remote access	Improved outreach via mobile units	Limited to urban and peri‐urban areas

*Source:* Author's elaboration.

### 4.3 Interaction Effects and Predictive Simulations

**FIGURE 2:**
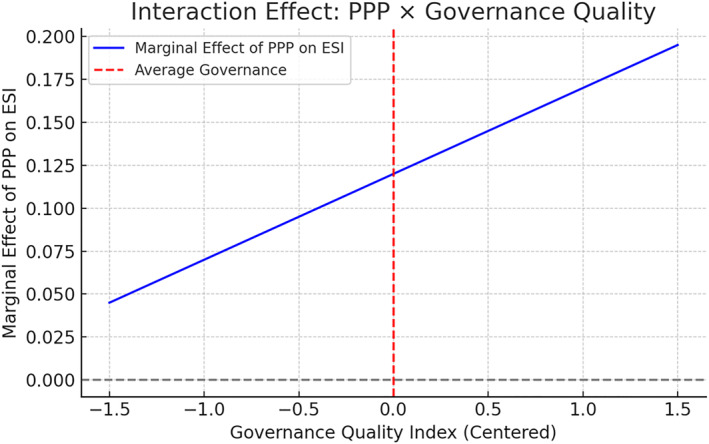
Interaction effect: Governance quality on PPP impact (economic sustainability index). *Source:* World bank PPI (ppi.worldbank.org), OECD health stats (oecd.org), WHO GHO (who.int/data/gho), EEA (eea.europa.eu), WGI (info.worldbank.org/governance/wgi), ISO (iso.org).

To evaluate moderation effects central to Research Question 2 (RQ2), we estimated interaction models that incorporate the Governance Quality Index as a moderator of PPP efficacy. Figure 2 illustrates how the marginal effect of PPP participation on the Economic Sustainability Index (ESI) increases with governance quality, confirming the hypothesis that well‐governed contexts enhance the sustainability impacts of PPPs.

Additional interaction terms, such as GDP per capita × PPP, were also tested but yielded less consistent patterns, suggesting that governance quality plays a more decisive moderating role in shaping sustainability outcomes.

**FIGURE 3:**
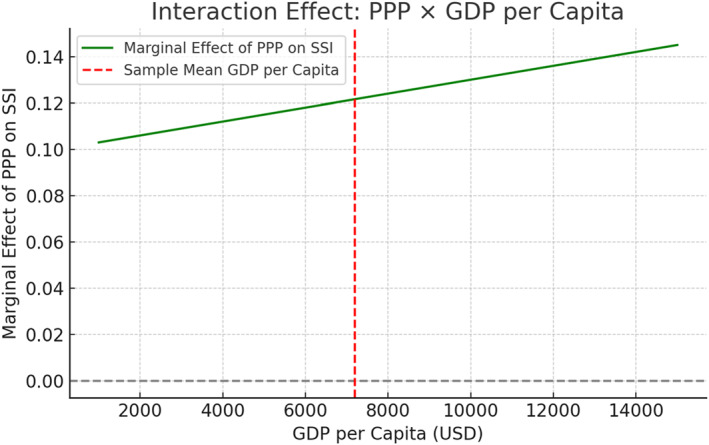
Interaction effect: GDP per capita on PPP impact (SSI). *Source:* World bank PPI (ppi.worldbank.org), WHO GHO (who.int/data/gho), OECD health stats (oecd.org), IMF [38].

A second interaction analysis was conducted to evaluate how the marginal effect of PPPs on the Social Sustainability Index (SSI) varies by GDP per capita. As shown below in Figure 3, higher income levels amplify the positive effect of PPPs on social inclusion outcomes, likely due to better infrastructure, regulatory systems, and fiscal capacity.

Supporting Information S2: Appendix 4 presents simulated marginal effects of PPPs on the economic sustainability index across varying levels of governance quality.

### 4.4 Multilayer Network Insights and Per‐Country Validation

This section combines structural and empirical insights from the multilayer network model to test the research hypotheses and examine cross‐country differences in health care PPP performance. Building on the framework presented in Section 3, three illustrative PPP projects—selected for their geographic diversity, variation in governance quality, and completeness of data—are analysed using key network metrics: degree centrality, betweenness centrality, and interlayer coupling.

A cumulative sustainability score, shown in Table 7, is then calculated by normalising and averaging these three indicators. The final column compares each project's performance against the expectations outlined in Hypotheses 1, 2, 3, offering a structured validation of the model's predictive relevance.

**TABLE 7:** Country‐level sustainability and hypothesis alignment.

Project ID/Country	Degree centrality	Betweenness centrality	Interlayer coupling	Cumulative sustainability score	Hypotheses alignment (1, 2, 3)
P01 (Kenya)	0.82	0.40	0.68	0.63	Moderate alignment (✓ 1, 2)
P02 (Bangladesh)	0.76	0.55	0.73	0.68	Strong alignment (✓ 1, 2, 3)
P03 (Peru)	0.65	0.38	0.60	0.54	Moderate alignment (✓ 1, 2)

*Source:* World Bank PPI, WHO GHO, OECD, EEA, and ISO 14001 compliance registers (2005–2022).

The cumulative sustainability score serves as a composite indicator of systemic integration, confirming that higher multilayer centrality is associated with stronger sustainability outcomes. Projects in Kenya (P01) and Bangladesh (P02) exhibit strong interlayer coupling and strategic centrality, aligning well with all three sustainability dimensions—economic, social, and environmental —thus supporting Hypotheses 1, 2, 3. Peru's project (P03), while slightly less integrated environmentally, still performs strongly on economic and social fronts, validating H1 and H2.

These results support the theoretical claim that multilayer integration enhances PPP resilience and performance. They also illustrate moderate cross‐country differences: Peru leads in raw sustainability scores, potentially due to stronger governance or implementation capacity, while Kenya and Bangladesh perform positively but more modestly. This reinforces the findings from RQ2, highlighting the role of institutional quality as a key moderator and underscoring the importance of tailoring PPP designs to local governance contexts for maximum sustainability impact.

## 5 Networked Stakeholder Architecture in Sustainable Health Care PPPs

Health care PPPs engage a diverse array of stakeholders, each pursuing distinct objectives, ranging from financial returns and regulatory compliance to social equity and environmental stewardship. In developing countries, achieving convergence across these interests is essential for sustainable outcomes. Stakeholders can be grouped along four key layers:

- Environmental stakeholders include green tech providers, environmental agencies, ministries of environment, NGOs, and waste management firms.
- Social stakeholders span frontline health care workers, local health councils, civil society groups, patient advocates, and marginalised communities.
- Governance stakeholders consist of PPP units, auditors, legal advisors, planning commissions, and oversight institutions.
- Economic and financial stakeholders encompass development banks, aid agencies, commercial lenders, investors, insurers, and public finance managers.

Effective collaboration among PPP stakeholders depends on structured approaches that align incentives and support shared goals. Multilayer network models help map these interactions by grouping stakeholders into financial, social, and governance layers. Cross‐cutting actors—such as ESG funds or data platforms—serve as connectors across layers, enhancing coordination. Artificial Intelligence strengthens this structure by enabling real‐time monitoring and performance optimization. These findings support RQ3, showing that integrated stakeholder networks contribute to more resilient and inclusive health care systems through better‐aligned PPP design.

**FIGURE 4:**
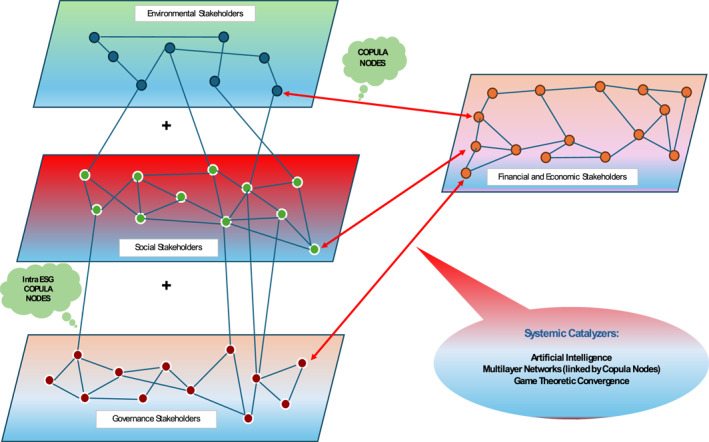
Enhanced multilayer network: PPP SC integrated with financial and external stakeholders. *Source:* Authors' compilation.

As shown in Figure 4, the model maps key actors across five layers—Environmental, Social, Governance, Economic, and Financial—illustrating how vertical project flows, peer coordination, and regulatory links interact. This structure not only reduces governance fragmentation but also supports sustainability through aligned stakeholder roles. It complements the econometric analysis by visualising how sustainability is co‐produced across domains. These insights reinforce RQ3 and Hypotheses H2 and H3, indicating that PPP success depends not only on contract design but also on coordinated governance and financing across ESG dimensions.

The empirical results not only validate the sustainability advantages of PPPs but also highlight governance and contractual mechanisms as levers that can translate these outcomes into actionable policy lessons.

## 6 Discussion and Policy Implications

Building on the empirical results presented in Section 4, this discussion situates the findings within the health care PPP literature and aligns them with the broader Sustainable Development Goals agenda.

This study set out to evaluate the sustainability performance of health care PPPs in emerging economies through an integrated multilayer network and econometric framework. The findings provide novel insights into how PPPs contribute to economic, social, and environmental outcomes, highlighting the systemic interdependencies that shape their long‐term success.

### 6.1 Economic and Social Dimensions

The results confirm that PPPs deliver superior economic performance compared with traditional procurement, consistent with earlier empirical work on health care PPPs that emphasised efficiency gains, structured risk allocation, and stronger financial discipline. Improvements in patient satisfaction and equity of access further suggest that PPPs can enhance social inclusion when governance quality is adequate. These findings resonate with recent evaluations of hospital PPPs in Latin America, Asia, and Africa, which underscore that user experience and community engagement are critical determinants of PPP legitimacy and sustainability (De Matteis [22]; Wibisono and Handoko [7]; Liu [25]). By linking these outcomes directly to SDG 3 (universal health coverage) and SDG 10 (reduced inequalities), the study confirms that PPPs can be important vehicles for advancing health care equity in low‐resource settings.

### 6.2 Environmental Dimension

While environmental gains are more modest, the positive effects observed align with emerging evidence that health care PPPs are beginning to integrate green procurement, energy efficiency standards, and waste management protocols (Cheng [23]; Xie [24]; OECD [27]). ISO 14001 compliance and renewable energy adoption are increasingly used to benchmark performance, but implementation remains uneven across emerging markets. These findings suggest that environmental outcomes require stronger regulatory incentives and contractual obligations if PPPs are to contribute meaningfully to SDGs 12 and 13 (responsible consumption and climate action).

### 6.3 Role of Governance

Governance quality emerged as a decisive moderator of sustainability outcomes, amplifying the benefits of PPPs across all three dimensions. This supports prior health care PPP studies that highlight the enabling role of robust institutions, clear accountability mechanisms, and transparent regulatory oversight (Roumboutsos and Saussier [33]; Kumar [9]). Where governance is weak, PPPs risk reproducing existing inequities or prioritising short‐term financial returns over long‐term system resilience. In contrast, contexts with stronger institutions demonstrate how contractual flexibility, resilience clauses, and participatory governance can reinforce systemic sustainability and align PPP design with SDG 16 (peace, justice, and strong institutions).

### 6.4 Methodological Contribution

Unlike most prior assessments of health care PPPs, which relied primarily on cost–benefit analysis [39], value‐for‐money tests, or single‐dimensional financial indicators (World Bank [40]; Carbonara and Pellegrino [16]), this study introduces multilayer network analysis to capture interdependencies across domains. By combining network metrics such as interlayer coupling and betweenness centrality with econometric panel models, the framework demonstrates how improvements in one domain (e.g., financial efficiency) can propagate to others (e.g., social inclusion). This systemic approach advances the methodological frontier of PPP evaluation, providing tools to identify synergies and trade‐offs that traditional linear models often overlook.

### 6.5 Policy Implications

The evidence suggests three key levers for policymakers and practitioners designing health care PPPs in emerging economies. First, embedding ESG‐based performance indicators into payment structures ensures alignment with sustainability objectives. Second, resilience clauses that anticipate systemic shocks—such as pandemics or fiscal crises—can protect both service delivery and equity. Third, the adoption of international benchmarks, such as the WHO UHC Index, strengthens transparency and comparability across projects. Collectively, these strategies can help governments and private partners co‐produce infrastructure that is financially viable, socially inclusive, and environmentally responsible.

In sum, this study demonstrates that health care PPPs, when supported by strong governance and designed with multidimensional sustainability in mind, can serve as a cornerstone of resilient health systems. By addressing long‐standing gaps in the literature and offering a replicable analytical framework, the findings provide actionable insights for advancing the Sustainable Development Goals through health care infrastructure partnerships.

## 7 Conclusion

In conclusion, this study integrates insights from health care PPP literature with new empirical evidence to demonstrate how multilayer approaches can advance both scholarly understanding and policy practice in line with the SDGs.

This study shows that health care PPPs are not only viable but also transformative tools for advancing sustainable infrastructure in emerging economies. By combining private sector efficiency, adaptive governance, and risk‐sharing mechanisms, PPPs consistently outperform traditional procurement models in financial sustainability, service accessibility, and patient satisfaction. While environmental gains are more limited, they become significant when embedded within integrated, ESG‐aligned strategies.

Utilising a novel multilayer network framework, the research underscores the profound interconnection among economic, social, and environmental outcomes. Projects with high system integration—‘multilayer hubs’—demonstrate superior resilience, efficiency, and equity. These insights support a transition from siloed evaluations to holistic policy design where incentives, governance, and contract structures reinforce one another.

As demographic pressures mount and health care inequalities persist, especially in low‐ and middle‐income countries, PPPs offer scalable, flexible solutions that align fiscal discipline with equity and sustainability. For future PPPs to succeed, models must be locally grounded, data‐driven, and responsive to context‐specific needs. Understanding how financial governance can catalyse improvements in access and continuity will be key to building inclusive and robust systems.

While this study provides a strong empirical foundation, it also recognises the need for stakeholder engagement. Future research should extend beyond emerging economies to test whether findings generalise to high‐income settings with different governance and financing structures. Additionally, incorporating perspectives from practitioners and communities will strengthen contextual relevance. Ultimately, effective PPPs require more than sound financing—they demand a collaborative, systems‐oriented approach that delivers enduring public value.

By incorporating these design principles, PPPs can become a cornerstone of resilient, inclusive, and sustainable health systems worldwide. The alignment of empirical evidence with the health care PPP literature and the SDG agenda provides a solid foundation for both scholarly inquiry and practical policy reform.

## Funding

The author has nothing to report.

## Ethics Statement

The author has nothing to report.

## Conflicts of Interest

The author declares no conflicts of interest.

## Supporting information


Supporting Information S1

Supporting Information S2

## Data Availability

The data that support the findings of this study are available on request from the corresponding author. The data are not publicly available due to privacy or ethical restrictions.
