# Supplementary material for: Sustainable Health Care Public‐Private Partnerships in Emerging Economies
Source: Int J Health Plann Manage. 2025 Oct 30;41(1):107–20. doi: 10.1002/hpm.70036 (PMC12794126; doi:10.1002/hpm.70036)
Supplement: Supplementary file 1 — Supporting Information S1 [file HPM-41-107-s001.docx]

**Supplementary Material**

**Mathematical Foundations for Multilayer Stakeholder Networks in Healthcare PPPs**

Multilayer network theory provides a comprehensive and mathematically precise framework (also considered in the Supplementary Material) for modeling dynamic, heterogeneous relationships among stakeholders in complex systems like Public-Private Partnerships (PPPs). Particularly in the healthcare sector of developing countries, where governance, social impact, environmental responsibility, and financial sustainability intertwine, multilayer networks capture the interdependencies and emergent behaviors that traditional models cannot fully represent.

Following Bianconi (2018) and Barabási (2016), a multilayer network is defined as a set of graphs:

*G = { G^{[α]} } for α = 1, ..., L*

Each layer represents a domain-specific network:

*G^{[α]} = (V^{[α]}, E^{[α]})*

Where V^{[α]} are the stakeholders and E^{[α]} are their connections within layer α.

Cross-layer interactions are enabled by interlayer edges and copula nodes defined as:

*v_i ∈ V^{[α]} ∩ V^{[β]} for α ≠ β*

To capture the temporal evolution of the network, we define the adjacency function:

*A_{ij}^{[α]}(t)*

**Key Mathematical Tools**

**1. Eigenvalue Spectrum Analysis**

Eigenvalue spectrum analysis evaluates the structural robustness of a multilayer network. The Laplacian matrix of a network layer, or the supra-Laplacian in multilayer systems, captures how well-connected the structure is. The second smallest eigenvalue, λ₂, called algebraic connectivity, quantifies the network's cohesion. A larger λ₂ implies greater resilience to disconnection or stakeholder dropout.

**2. Multilayer Modularity Optimization**

Modularity optimization identifies community structures within and across network layers. These communities are functional clusters of stakeholders with stronger internal than external connections. Modularity is quantified through a function Q, maximizing which helps detect latent coalitions and policy-relevant groupings across ESG and financial domains.

To detect community structure across layers:

*Q = (1 / 2μ) ∑_{ijαβ} [ A_{ij}^{[αβ]} - γ^{[αβ]} * (k_i^{[α]} * k_j^{[β]} / 2m^{[αβ]}) ] δ(c_i, c_j)*

**3. Interlayer Metrics**

Interlayer metrics measure how stakeholders and interactions span across multiple domains. Key indicators include:

- Overlap Degree: Represents the number of layers in which a stakeholder is active, signaling their strategic cross-domain influence.
- Mutual Information: Measures the shared information between two layers, indicating how closely aligned different domains are in their stakeholder structures.

Overlap degree of stakeholder i:

*o_i = | { α : v_i ∈ V^{[α]} } |*

Mutual information between two layers α and β:

*I(α; β) = ∑_{x, y} p_{αβ}(x, y) * log( p_{αβ}(x, y) / (p_{α}(x) * p_{β}(y)) )*

**Game Theory Applications**

Cooperative game theory models strategic interactions where stakeholders can form coalitions to maximize joint outcomes. Three central tools enable fair and stable outcomes:

1. Shapley Value: Determines each stakeholder’s average marginal contribution across all coalition possibilities. This ensures fair allocation of collective benefits.

*ϕ_i(v) = ∑_{S ⊆ N \ {i}} (|S|! * (n - |S| - 1)! / n!) * [ v(S ∪ {i}) - v(S) ]*

1. Nash Bargaining Solution: This solution captures a fair division of outcomes between parties, assuming rational negotiation and disagreement fallback values.

*x* = arg max_{x ∈ X} ∏_{i=1}^n (x_i - d_i)*

1. Core of the Game: Defines the set of payoff distributions where no subgroup of stakeholders would prefer to break away and form an alternative coalition.

*Core(v) = { x ∈ ℝ^n : ∑_{i ∈ N} x_i = v(N), ∑_{i ∈ S} x_i ≥ v(S) ∀ S ⊆ N }*

**AI-Driven Optimization**

Graph Neural Network update for node i at layer l+1:

*h_i^{(l+1)} = σ( ∑_{α} ∑_{j ∈ 𝒩^{[α]}(i)} W^{[α]} h_j^{(l)} + b^{[α]} )*

Reinforcement Learning Q-function:

*Q*(s, a) = max_{π} 𝔼[ r_t + γ Q*(s_{t+1}, π(s_{t+1})) ]*

These analytical tools provide a unified platform for modeling interdependencies, optimizing stakeholder decisions, and dynamically adjusting policies to enhance PPP sustainability in healthcare systems.

This framework enables strategic, data-driven governance of complex healthcare PPPs by combining multilayer network theory with cooperative game models and AI-driven learning systems. It equips decision-makers with the mathematical and technological tools required to ensure inclusiveness, resilience, and adaptive efficiency across domains.

**References**

Barabási, A. L. *Network science* (Vol. 3). Cambridge, UK:: Cambridge University Press. 2016.

Bianconi G. Multilayer Networks: Structure and Function. Oxford University Press; 2018.
