# Supplementary material for: Sustainable Health Care Public‐Private Partnerships in Emerging Economies
Source: Int J Health Plann Manage. 2025 Oct 30;41(1):107–20. doi: 10.1002/hpm.70036 (PMC12794126; doi:10.1002/hpm.70036)
Supplement: Supplementary file 2 — Supporting Information S2 [file HPM-41-107-s002.docx]

**Appendix**

**1. Supplemental Health Sector-Specific Performance Indicators**

To address the need for more concrete healthcare system relevance, the following subsection shows indicators that capture hospital-level outcomes aligned with the social sustainability dimension.

While the composite indices (ESI, SSI, EnSI) provide a macro-level view of sustainability, this subsection introduces direct hospital performance indicators to improve relevance to healthcare-specific policy. Four additional metrics were extracted from secondary datasets available for 72 of the 148 projects, covering the period 2008–2021. These metrics include:

- Hospital readmission rates (within 30 days)

- Average surgical wait time (in days)

- Bed occupancy rate (%)

- Emergency response time (in minutes)

These indicators were normalized using z-scores and integrated into the social sustainability regressions. They were used to validate whether PPP-managed facilities outperformed traditional public projects in direct service delivery outcomes.

TABLE A.1 presents the comparative performance across PPP and traditional procurement models.

| **Indicator** | **PPP Mean** | **Traditional Mean** | **Standard Deviation** | **p-value (t-test)** |
| --- | --- | --- | --- | --- |
| Hospital Readmission Rate (%) | 12.4 | 16.8 | 3.2 | 0.008 |
| Surgical Wait Time (days) | 9.2 | 14.5 | 4.7 | 0.011 |
| Bed Occupancy Rate (%) | 83.5 | 71.2 | 5.9 | 0.003 |
| Emergency Response Time (min) | 11.8 | 18.6 | 6.1 | 0.001 |

**Sources:** WHO GHO ([who.int/data/gho](https://www.who.int/data/gho)), OECD ([oecd.org](https://www.oecd.org)), World Bank PPI ([ppi.worldbank.org](https://ppi.worldbank.org)).

**2. Clarifying Multilayer Metrics in Healthcare PPPs**

This appendix offers a plain-language explanation of multilayer network metrics used in the analysis of Public-Private Partnerships (PPPs) in healthcare systems. It is designed to help policy-makers and practitioners understand how complex network analytics translate into actionable planning and contract design decisions. Multilayer network metrics provide insights into how changes in one aspect of a health system (e.g., budget allocation) can affect other outcomes (e.g., equity of access). For instance, alleviating financial pressures in a hospital may lead to improvements in service quality and patient retention across the network. This is modeled as a cross-domain interaction or 'interconnected result'. Practical policy applications include adjusting PPP contract length based on systemic fragility or embedding resilience incentives in weaker areas of network connectivity.

TABLE A.2. Interpreting Multilayer Metrics for Policy Design

| Metric | Plain-Language Meaning | Real-World Analogy | PPP Design Application |
| --- | --- | --- | --- |
| Interlayer Coupling | Measures how decisions in one domain (e.g., finance) influence outcomes in another (e.g., access). | Such as how hospital budget decisions affect patient wait times and service access. | Design bundled contracts integrating infrastructure and social outcomes. |
| Multilayer Centrality | Identifies key actors or hospitals influential across all domains. | Like a central train station connecting multiple metro lines—a strategic node. | Prioritize high-centrality hospitals for phased investments. |
| Network Robustness | Reflects a system's resilience to disruption or failure. | Comparable to a bridge that remains intact even if one pillar fails. | Include contingency clauses and emergency response protocols in contracts. |

**Sources: author elaboration**

**3 Robustness Analyses**

A comprehensive suite of diagnostic checks was performed to assess the validity, consistency, and internal robustness of the empirical findings reported in Section 4.2. These robustness analyses ensure that the observed relationships between PPPs and multidimensional sustainability outcomes are not artifacts of modeling assumptions, sample composition, or data irregularities. All computations are based on harmonized panel data spanning 22 emerging economies from 2005 to 2022, sourced from:

- World Bank Private Participation in Infrastructure (PPI) Database (PPP identifiers, financial structure),
- OECD Health Statistics (healthcare quality and accessibility),
- WHO Global Health Observatory (social inclusion metrics),
- European Environment Agency (EEA) and ISO 14001 data (environmental indicators),
- World Bank Worldwide Governance Indicators (governance quality, legal origin classification).

Three index formulations were tested: (1) Equal Weights (simple average of normalized components), (2) Principal Component Analysis (PCA) to extract the dominant latent factor, and (3) Expert-derived weighting based on a Delphi survey of development professionals. Across all three formulations, the coefficient on the PPP dummy remained positive and statistically significant in two-way fixed effects models, reinforcing index validity.

Regression models were estimated separately for low-income and middle-income country subsamples, as defined by the World Bank Atlas method. PPP effects were notably stronger in the middle-income group across all sustainability indices, suggesting the moderating influence of institutional maturity. Coefficients on PPP dummy were up to 30% larger in magnitude in middle-income samples.

To mitigate potential endogeneity in PPP project allocation, legal origin was used as an instrument (coded as 1 = common law, 0 = civil law). First-stage regressions confirmed strong instrument relevance (F-statistics > 10). Second-stage estimates corroborated baseline effects, with PPP dummy coefficients increasing by 8–12% relative to OLS, supporting a causal interpretation.

To detect possible spatial autocorrelation, both Spatial Lag Models (SLM) and Spatial Error Models (SEM) were estimated. A spatial weight matrix based on inverse geographic distance was applied. Spatial rho and lambda parameters were small (< 0.05) and statistically insignificant (p > 0.25), suggesting no spatial clustering of residuals or unobserved common shocks.

TABLE A.3. Numerical Summary of Robustness Checks

| **Robustness Test** | **Methodology** | **PPP Coefficient Range** | **Significance Level** | **Implication** |
| --- | --- | --- | --- | --- |
| Alternative Index Specifications | Equal Weights, PCA, Expert Weights | 0.148 – 0.162 | p < 0.01 | Results are stable across formulations |
| Subsample Analyses | Separate regressions by income group | 0.142 (low) – 0.186 (middle) | p < 0.05 | Stronger PPP effects in middle-income countries |
| Instrumental Variable (IV) Estimation | Legal origin as an instrument (2SLS) | 0.163 – 0.170 | p < 0.01 | Causal inference confirmed |
| Spatial Econometric Models | SLM & SEM using geographic distance matrix | 0.150 – 0.153 | p < 0.05 | No spatial dependence detected |

**Sources: World Bank PPI (**[**ppi.worldbank.org**](https://ppi.worldbank.org)**), WHO GHO (**[**who.int/data/gho**](https://www.who.int/data/gho)**), OECD (**[**oecd.org**](https://www.oecd.org)**), IMF IFS (**[**data.imf.org**](https://data.imf.org)**).**

In sum, all robustness strategies converged on consistent empirical findings, reinforcing the credibility of the reported PPP effects across economic, social, and environmental dimensions. These results support the study’s multilayer network approach and confirm the systemic validity of using integrated sustainability metrics in assessing PPP performance in emerging economies.

**4. Predictive margins**

Confidence intervals are computed using robust standard errors clustered at the country level. The margins indicate increasing marginal returns to PPPs in high-quality governance environments.

TABLE A.4 – Predictive margins

| Governance Index | Marginal Effect (PPP on ESI) | 95% CI Lower | 95% CI Upper |
| --- | --- | --- | --- |
| -1.5 | 0.045 | 0.01 | 0.08 |
| -0.5 | 0.095 | 0.05 | 0.14 |
| 0.0 | 0.12 | 0.075 | 0.165 |
| 0.5 | 0.145 | 0.1 | 0.19 |
| 1.5 | 0.195 | 0.15 | 0.24 |

**Sources: World Bank PPI (**[**ppi.worldbank.org**](https://ppi.worldbank.org)**), OECD (**[**oecd.org**](https://www.oecd.org)**), WHO GHO (**[**who.int/data/gho**](https://www.who.int/data/gho)**), EEA (**[**eea.europa.eu**](https://www.eea.europa.eu)**).**
